# Computational Study of Helicase from SARS-CoV-2 in RNA-Free and Engaged Form

**DOI:** 10.3390/ijms232314721

**Published:** 2022-11-25

**Authors:** Francesca Di Matteo, Giorgia Frumenzio, Balasubramanian Chandramouli, Alessandro Grottesi, Andrew Emerson, Francesco Musiani

**Affiliations:** 1 Laboratory of Bioinorganic Chemistry, Department of Pharmacy and Biotechnology, University of Bologna, Viale G. Fanin 40, 40127 Bologna, Italy; 2 Super Computing Applications and Innovation, Department HPC, CINECA, via Magnanelli 6/3, 40033 Casalecchio di Reno, Italy; 3 Department HPC, CINECA, via dei Tizii 6, 00185 Roma, Italy

**Keywords:** SARS-CoV-2, NSP13, helicase, RNA, molecular dynamics, HPC

## Abstract

Severe acute respiratory syndrome coronavirus 2 (SARS-CoV-2) is the causative agent of the pandemic that broke out in 2020 and continues to be the cause of massive global upheaval. Coronaviruses are positive-strand RNA viruses with a genome of ~30 kb. The genome is replicated and transcribed by RNA-dependent RNA polymerase together with accessory factors. One of the latter is the protein helicase (NSP13), which is essential for viral replication. The recently solved helicase structure revealed a tertiary structure composed of five domains. Here, we investigated NSP13 from a structural point of view, comparing its RNA-free form with the RNA-engaged form by using atomistic molecular dynamics (MD) simulations at the microsecond timescale. Structural analyses revealed conformational changes that provide insights into the contribution of the different domains, identifying the residues responsible for domain–domain interactions in both observed forms. The RNA-free system appears to be more flexible than the RNA-engaged form. This result underlies the stabilizing role of the nucleic acid and the functional core role of these domains.

## 1. Introduction

A novel coronavirus (severe acute respiratory syndrome coronavirus 2, SARS-CoV-2) has been identified as the pathogen responsible for the outbreak of a severe, rapidly developing pneumonia, coronavirus disease 2019 (COVID-19) [1]. The first event of the SARS-CoV-2 emergency occurred when a group of patients at hospitals in Wuhan, China, reported cases of pneumonia with an unknown cause. The symptoms were characterized by fever, pneumonia, cough, and dyspnea. The first known case can be dated to 8 December 2019, while the international state of emergency was announced by the WHO on 30 January 2020. On 11 March in the same year, the World Health Organization (WHO) defined COVID-19 as a pandemic [2]. Up to now, the COVID-19 disease has caused more than 6 million deaths worldwide. The novel coronavirus belongs to the severe acute respiratory syndrome-related coronavirus species, which arises from the genus Betacoronavirus, a genus that infects only mammals, from the family *Coronaviridae*. The family belongs to the order Nidovirales, which includes viruses with single-stranded RNA genomes of positive polarity [3]. The virus, as far as humans are concerned, is responsible for respiratory infections that manifest themselves as a mild to severe clinical picture [1]. The beta-coronavirus group also includes severe acute respiratory syndrome coronavirus (SARS-CoV) and Middle East respiratory syndrome coronavirus (MERS-CoV), which emerged in 2002 and 2012, respectively, which caused severe respiratory diseases with high pathogenicity [3].

**Figure 1:**
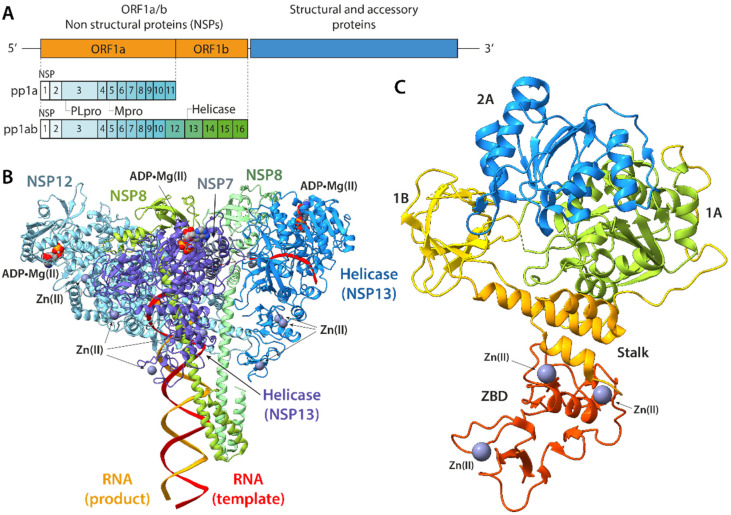
(**A**) Organization of the SARS-CoV-2 genome. The RNA encodes two categories of proteins: non-structural proteins (NSPs) and structural and accessory proteins. NSPs are encoded in ORF1a and ORF1b. Cap-dependent translation begins at ORF1a and produces pp1a, which encompasses NSP1–11, or pp1ab, which encompasses NSP12–16. (**B**) Ribbon diagram of the RdRp-(NSP13)_2_ supercomplex structure in the RNA-engaged form (PDB ID: 7RDY). The NSP12 (RdRp), NSP7, and NSP8 subunits are in light blue, gray, and green, respectively. The two NSP13 subunits are in blue and violet for the RNA-engaged and the RNA-free form, respectively. The template and the product RNA strands are in red and orange, respectively. Zn(II) and Mg(II) ions, as well as ADP molecules are reported as spheres colored according to the atom type. (**C**) Ribbon diagram of NSP13 in the RNA-free form (PDB ID: 6ZSL). The ribbons are colored in red, orange, yellow, green, and blue to highlight the position of the ZBD, Stalk, 1B, and 1A and 2A domains, respectively. Zn(II) ions are reported as purple spheres.

The coronavirus virion is defined by four structural proteins: spike, envelope, membrane, and nucleocapsid [4,5]. The latter covers the genome, which consists of a positive-sense single-stranded RNA of approximately 30 kilobases [6]. This, moreover, is enclosed by a lipid bilayer formed by the abovementioned membrane proteins, namely the spike, the membrane, and the envelope [4,5]. All of these are involved in the virus’s entry into the cell. The recognition of and the binding to the host cell is the responsibility of the spike protein [7], which binds to the target cell through specific interactions with cellular receptors [8]. Once the virus has entered the host cell, the viral genome is released into the cytoplasm, and a finely regulated system is triggered (Figure 1A). The cell’s ribosomes are called up and the viral RNA, which is capped at 5′ and polyadenylated at 3′, assists in the translation of two replicases, ORF1a and ORF1b, which represent as much as two-thirds of the viral genome [1]. The translation generates two large polyproteins, pp1a and pp1ab, which are 4405 and 7096 amino acids long, respectively [1,4,8]. Sixteen non-structural proteins are then co- and post-translationally released from pp1a and pp1ab. Specifically, pp1a produces non-structural proteins (NSPs) 1–11, while pp1ab produces NSP1 to NSP16. This is a ribosomal frameshift present on a short overlap of ORF1a and ORF1b. Indeed, the production of a polyprotein is determined by whether the stop codon on ORF1 is recognized or ignored [1]. If the stop codon is ignored, there is a-1 ribosomal frameshift in the overlapping region, allowing the production of the larger polyprotein, pp1ab [8]. The stoichiometric balance between pp1a and pp1ab is tipped towards pp1a, which is about 1.4 times more expressed than pp1ab. However, it is in the pp1ab polyprotein that the heart of the replication and transcription machinery is contained. The individual NSPs are then derived from proteolytic cleavage on pp1a and pp1ab operated by the papain-like protease (PLpro), which is encoded by NSP3, and the chymotrypsin-like or main protease (Mpro), encoded by NSP5 [8].

Even though the roles of some NSPs remain obscure, most of the products of pp1a and pp1ab’s proteolysis give rise to the viral replication–transcription complex (RTC) within organelles drafted by NSP3, NSP4, and NSP6 [8,9,10]. The RTC resides in convoluted membranes from the endoplasmic reticulum. Its main tasks are those of replication, which involves the synthesis of a negative strand of RNA—complementary to the positive strand of departure—which acts as a template for the progeny of positive strands, and the synthesis of sub-genomic mRNAs, characterized by discontinuous transcription [8,11]. The minimal RTC complex, forming an RNA-dependent RNA polymerase (RdRp), is formed by the catalytic subunit (NSP12), at least one NSP7 subunit, and two copies of NSP8 [8,12,13,14,15]. The RdRp is then elongated by two subunits of the NSP13, a 67 kDa helicase belonging to the SF1 family of the 1B helicase superfamily [8,13,16,17,18], which is the subject of this study.

NSP13 performs its function by utilizing the energy released from the hydrolysis of a molecule of nucleotide triphosphate to catalyze the unwinding of the DNA or RNA double-stranded molecule before the presence of an overhang at the 5′ filament, which shows that its helicase activity occurs in a 5′–3′ direction [19]. For this particular helicase, both substrates can be accepted, and it has been shown that, in vivo, there is robust enzymatic activity with RNA, but, in vitro, there is an equally strong processivity with DNA [19]. In a bulk unwinding assay, it was observed that DNA unwinding was a faster event than that of RNA, thus showing better activity with DNA as a substrate [20]. Another study suggested that RNA is preferred as a substrate for unwinding if high concentrations of ATP are present [21].

The structure of the SARS-CoV-2 RdRp-(NSP13)_2_ supercomplex in the RNA-engaged form was resolved at the end of 2021 at 3.10 Å resolution by cryo-electron microscopy (Figure 1B) [22]. In the RTC, NSP13 interacts with the other NSPs of the RTC through interactions between its *N*-terminal ZBD and the *N*-terminal region of each NSP8 unit [18]. Interestingly, in only one of the two NSP13 subunits which form the supercomplex, a single-stranded RNA fragment was found to be bound to the protein, while the second NSP13 subunit was in the RNA-free form. An ADP molecule coordinated with a Mg(II) ion was found to be bound to each NSP13 subunit as well as to the NSP12 subunit. Moreover, each NSP13 subunit binds three Zn(II) ions, while the NSP12 subunit binds a single Zn(II) ion.

The high-resolution structure of NSP13 was resolved in 2020 at 1.94 Å resolution in the RNA- and ADP-free form (Figure 1C) [19]. From a structural point of view, NSP13 exhibits the hallmarks of the superfamily to which it belongs. It consists of five domains. The first is one of the most conserved domains in the *Nidovirales* order, which is the *N*-terminal zinc binding domain (ZBD, Residues 1–100) that coordinates three Zn(II) ions; a helical “Stalk” domain (101–150); a β-barrel domain (1B, 151–261); and two “RecA-like core helicase domain” subdomains (namely 1A and 2A, Residues 262–442 and 443–596, respectively), which can be referred to as the catalytic core of the helicase, since it holds the residues known to be responsible for nucleotide binding and hydrolysis [19].

Although NSP13 is a highly conserved protein—in fact, it differs from SARS-CoV by only one amino acid [23]—its exact role in the viral cycle has not yet been delineated; in addition, it is a potential and interesting drug target [23]. The structures of NSP13 in the RNA-free and in the RNA-engaged form are nearly identical in their general folding and in the domains’ composition. However, the domains are found in slightly different positions (Figures S1 and S2 in the Supporting Information) because of the presence of the RNA fragment. In particular, in the RNA-engaged form, Domain 1B moves slightly away from the other domains with which it is in contact in the RNA-free form to make room for the single strand of RNA that runs through the entire structure of the protein. Domain 2A is also positioned slightly differently from the other domains in the two forms. This movement is also attributable to the presence of the RNA fragment and the presence of an ADP molecule in a pocket at the interface between Domains 1A and 2A. These rearrangements cause an overall change in the structure, so that the root mean square deviation (RMSD) between the Cα of the two experimental structures is 0.32 nm.

The aim of this work was to investigate NSP13 from a structural and dynamical point of view by taking advantage of the recent structures of the protein in the RNA-free [19] and in the RNA-engaged form [22], the latter of which was extracted from the RdRp-(NSP13)_2_ supercomplex. Therefore, two 10-μs-long sets of atomistic molecular dynamics simulations were implemented to examine the different conformational behaviors of NSP13 separated by the RTC complex in the presence or in the absence of an RNA fragment at the microsecond timescale. In addition to the classical structural observations, it was deemed interesting to explore the specific contact frequencies occurring between the single helicase domains. In other words, emphasis was placed on the residues involved in the mutual interactions of individual domains. The latter analysis was carried out in the aim of obtaining detailed information on the individual residues that change their interaction partners from the RNA-free to the RNA-engaged form of the SARS-CoV-2 helicase.

## 2. Results and Discussion

### 2.1. General Behaviour of the RNA-Free and the RNA-Engaged Systems

The structural stability of the two helicase systems was tested during the simulations by calculating the deviations of each structure from the starting coordinates and the fluctuation of each residue with respect to the average structure after superimposition on the Cα atoms.

#### 2.1.1. Deviations from the Starting Structure

**Figure 2:**
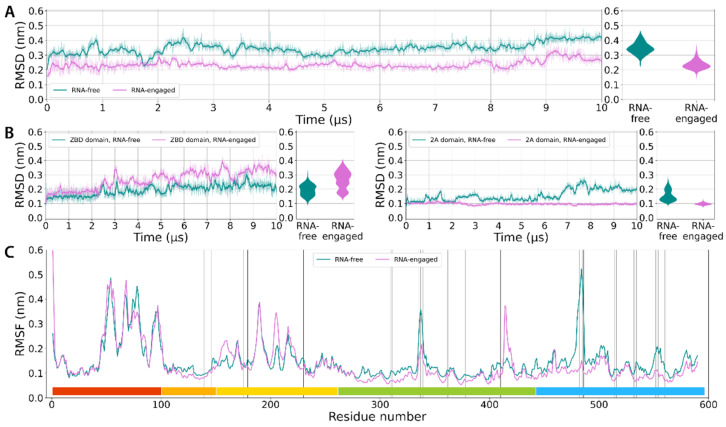
Root mean square deviations (RMSD) of NSP13 from the initial structures plotted as a function of the simulation time calculated for the entire system (**A**) and for selected domains (**B**). RMSD values for NSP13 in the RNA-free and in the RNA-engaged form are in dark cyan and orchid, respectively. The bold lines were obtained by applying a Savitzky–Golay filter [24] in order to cut out the noise. To the right of each plot, a violin representation of the RMSD distribution is provided. (**C**) Root mean square fluctuations values as a function of the residue number for NSP13 in the RNA-free (dark cyan) and RNA-engaged (orchid) form. The vertical lines highlight the position of the residues that are within 0.35 nm from the RNA strand in the RNA-engaged starting structure, while the horizontal bars report the position of each domain in the sequence and are colored according to the domain coloration in Figure 1C.

The RMSD for the Cα atoms of the whole protein was calculated and is depicted in Figure 2A. The behavior of the RMSD as a function of the simulation time showed that both the RNA-free and the RNA-engaged systems relax to conformations that differ by around 0.34 and 0.22 nm from the original system, respectively. For both simulations, the plateau of the structural drift was reached within the first microsecond. Notably, the RMSD of the RNA-free form was slightly higher than that of the RNA-engaged form. In other words, it appears that the presence of the RNA fragment and of the ADP molecule provides some additional stability to the NSP13 protein. In addition to the total RMSD, the deviations belonging to each domain were also calculated (see Figure 2B and Figures S2–S5 in the Supporting Information). The largest differences between the RNA-free and the RNA-engaged form were observed in the case of the ZBD and 2A domains. In the case of the ZBD domain (Figure 2B), the system appeared to show higher stability in the case of the RNA-free system, while the RNA-engaged system showed RMSD values that often oscillated between ~0.30 nm and up to more than 0.40 nm throughout the trajectory. This behavior, which could be due to the active contribution of the ZBD domain in RNA processing, was not affected even by excluding the residues at the *N*-terminal from the RMSD calculation. The coordination geometries of the three Zn(II) ions did not change during both simulations. The 2A domain remained extremely stable in the RNA-engaged form (Figure 2B), while in the RNA-free form, it experienced higher structural variability. The latter result can be ascribed to the presence of both the RNA fragment, which pushes Domain 1A towards Domain 2A, and the presence of the ADP molecule coordinated by the Mg(II) ion, which stabilises the interaction between Domain 1A and Domain 2A, resulting in a reduction in the fluctuations of the latter. 

#### 2.1.2. Backbone Fluctuations

The residue-based root mean square fluctuation (RMSF) of the trajectories was calculated for the Cα atoms to measure the flexibility of the protein chain (Figure 2C). The data show that there are different RMSF in specific regions of the protein. Residues 50 to 100, which belong to ZBD, possess higher flexibility. Residues 180 to 220 also show significant variation, indicating a certain flexibility of the 1B domain to which they belong. This is an expected result, considering the poorness in the secondary structural elements of the latter domains and the presence of long loops. Remarkably, in the RNA-free form, there are two clearly visible RMSF peaks, one around the region of Residues 335–339 and another near the region of Residues 482–486. These areas belong to two loops located in Domains 1A and 2A that are in direct contact with the RNA strand in the RNA-engaged starting structure. Moreover, the nucleotide binding site is mainly located in a cleft between Domains 1A and 2A. This explains the presence of the RMSD peaks in the structure when the RNA is not present, unlike the system occupied by the nucleic acid, which has considerably lower RMSF values in these regions due to the RNA’s stabilizing effect. On the other hand, the loop formed by Residues 204–208 is more flexible in the RNA-engaged form due to a displacement of the 2A domain, of which such a loop is part, due to insertion of the RNA. This movement of the 2A domain causes the breaking of some interactions with Domain 1A stabilizing the 204–208 loop in the RNA-free form (see also Section 2.3.1 below). Moreover, the 415–420 region experiences larger fluctuations in the RNA-engaged form. This is because of the absence of a small α-helix in the RNA-free form (Residues 419–421) that is not present any longer in the RNA-engaged form, possibly due to a conformational rearrangement that is necessary to make room for the nucleic acid. The absence of this helix causes major fluctuations in the 415–420 region, which lacks secondary structural elements and is exposed to the solvent in the RNA-engaged form. Other regions do not show significant RMSF differences within the two systems, apart for the *N*-terminal residues.

#### 2.1.3. Clustering

**Figure 3:**
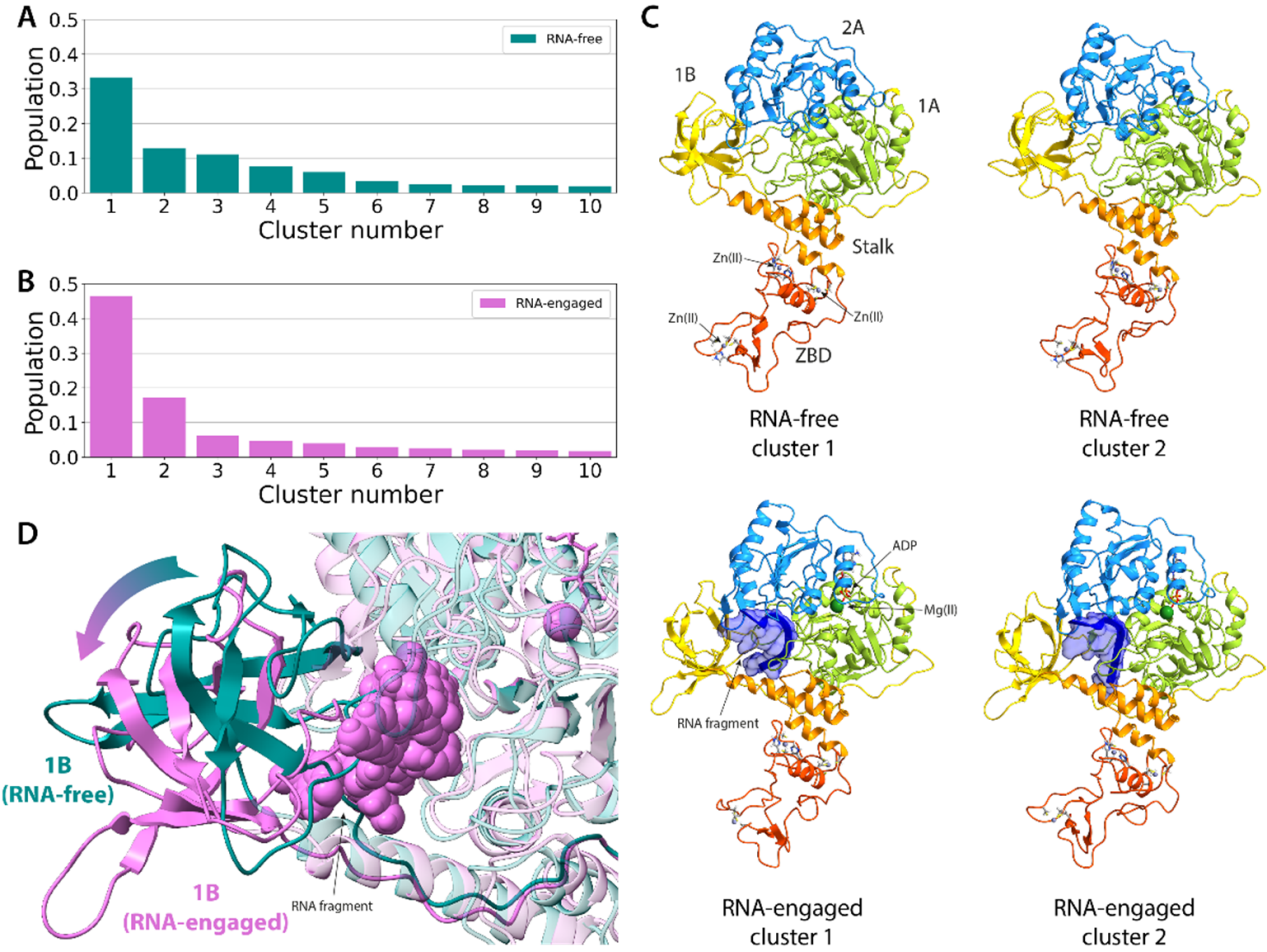
Cluster analysis of the trajectories of NSP13. Panels (**A**,**B**) show the bar plots of the cluster’s relative population in the RNA-free (dark cyan) and RNA-engaged (orchid) form, respectively. (**C**) Representative structures of the two most populated clusters in each system are shown as ribbons colored as in Figure 1C. The Zn(II) and Mg(II) ions are reported as spheres, while the Zn(II) coordinating residues are shown as sticks. All the atoms are colored according to the atom type. The RNA fragment is displayed as blue ribbons and the molecular surface is shown in light blue. (**D**) Details of the displacement of the 1B domain passing from the RNA-free (dark cyan ribbons) to the RNA-engaged (orchid) form. The arrow indicates the direction of the conformational rearrangement. The RNA atoms are shown as orchid-colored spheres.

A cluster analysis was performed for both the RNA-free and the RNA-engaged system in accordance with the GROMOS algorithm [25]. For each trajectory, 10,000 frames were analyzed and clustered using a cutoff of 0.17 nm. For the RNA-free system, 109 clusters were found (Figure 3A), with approximately 65% of the frames contained into the first four clusters. The trajectory of the RNA-engaged system was clustered in only 62 clusters instead, with ~64% of the frames contained in the first two clusters (Figure 3B). Thus, it appears that the presence of the nucleic acid induces a lower conformational variability in the structure of NSP13, in line with what has been observed in the RMSD and RMSF analyses.

The main difference in the conformation of the representative structure of the first two clusters in both systems (Figure 3C) is the relative position of the ZBD domain with respect to the other domains, along with other minor conformational changes. A comparison of the most representative structures in the most populated clusters in the RNA-free and RNA-engaged form of NSP13 showed a difference in the relative position of the ZBD domain with respect to the other domains and also a variation in the position of the 1B and 2A domains that can be ascribed to the presence of the single-stranded RNA fragment. Notably, Domain 1B is translated by about 5–7 Å and rotates about 30° clockwise to give space to the nucleic acid fragment (Figure 3D). The remaining regions of the proteins are almost perfectly superimposable.

### 2.2. Collective Motions in the RNA-Free and RNA-Engaged Forms

#### 2.2.1. Correlation of the Motions

**Figure 4:**
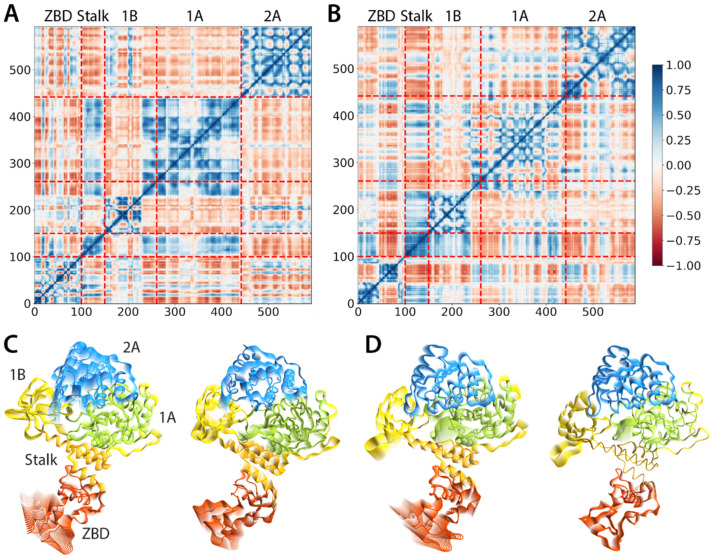
Residue–residue map of the Cα covariance maps of RNA-free (**A**) and RNA-engaged (**B**) NSP13. Blue regions show positive correlations, while dark red regions indicate anticorrelations. The red dashed lines indicate the border between subsequent domains. The bottom panels report the structural variations occurring in the first two principal components (PC) in RNA-free (**C**) and RNA-engaged (**D**) NSP13. In the bottom panels, NSP13 ribbons are colored as in Figure 1C.

Motion correlations between various sub-parts of NSP13 can be characterized by a calculation of the covariance matrices of the amino acids’ displacements. The corresponding maps computed using the coordinates of the Cα atoms are shown in Figure 4A and 4B for the RNA-free and RNA-engaged forms, respectively. NSP13 shows a slightly different dynamic behavior in the absence or in the presence of the RNA fragment. In particular, the ZBD domain appears to move as a single entity in the RNA-free form, while it appears to behave as two sub-domains of ~50 residues each, with a negative motion correlation, meaning that they move in opposite directions. Moreover, in the RNA-free form, the motion of the ZBD domain is anticorrelated with that of the Stalk, 1A, and 2A domains and is weakly correlated with the 1B domain. The ZBD domain in the RNA-engaged form shows a more complex motion with respect to the other domains because of the division into two sub-domains. The Stalk domain’s motion is correlated with that of the 1A domain while it is anticorrelated to that of the 2A domain in both systems. On the other hand, the Stalk domain’s motion appears to be anticorrelated to that of Domain 1B in the RNA-free form, while the presence of the RNA fragment induces a strong Stalk–1B motion correlation. The motion of the 1B domain appears to be anticorrelated with respect to both the 1A and 2A domains. Finally, Domain 1A’s motion is always anticorrelated with respect to the motion of the 2A domain, even if the presence of the RNA fragment seems to weaken this correlation of the motions.

To summarize, the main differences observed in the covariance maps are as follows: (i) the ZBD moves as a single entity in the RNA-free form, while is divided in two sub-domains experiencing a reciprocal anticorrelated motion in the RNA-engaged form; (ii) the Stalk domain’s motions are anticorrelated with the 1A domain in the RNA-free form and are correlated in the RNA-engaged form; and (iii) the collective intradomain motions of Domain 1A are less correlated in the RNA-engaged form, meaning that the domain moves less as a single entity. The exact opposite can be observed for the Stalk domain, the motions of which appear to be more concerted in the presence of the RNA fragment.

#### 2.2.2. Principal Component Analysis

In order to characterize the main motions characterizing NSP13 in the two different forms studied in this work, the two trajectories were further analyzed using principal component analysis (PCA). In general, the dynamic behavior of NSP13 in both states could can be classified into at least 20 collective motions without any one of them being truly predominant over the others. The projection of the first two principal components, PC1 and PC2, on the structures of the RNA-free and RNA-engaged NSP13 was then analyzed. In the case of the RNA-free NSP13 (Figure 4C), the first two PCs accounted for 26% and 14% of the variance. The motion of PC1 could be mainly described as the swing of the ZBD domain with respect to the 2A domain, while PC2 corresponded to a general rearrangement of all the domains without any predominant motion being noticed. In the RNA-engaged NSP13 (Figure 4D), the projection of PC1 (accounting for 19% of the variance) again revealed a swinging motion of the ZBD domain, but this time toward Domains 1B and 1A. Finally, in the case of the PC2 projection of the RNA-engaged form (12% of variance), it was possible to observe how the Stalk domain remained immobile by acting as a fulcrum while the other domains underwent a structural rearrangement mainly involving the ZBD and 1B domains.

#### 2.2.3. Conformational Space

**Figure 5:**
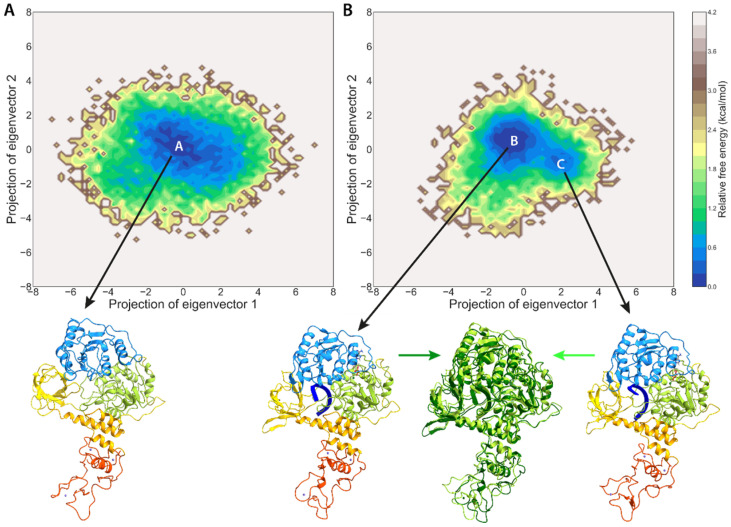
Free energy surface obtained from the 10-μs-long MD trajectories projected on the first two principal components of the Cα position’s covariance matrix for RNA-free (**A**) and RNA-engaged (**B**) NSP13. The surfaces are colored according to the relative free energy. Representative structures from the more populated regions are reported in the bottom panels for the single highly populated region of RNA-free NSP13 (Basin A, left panel) and RNA-engaged NSP13 (panels on the right). Of the three right-hand panels, the leftmost one and the rightmost one represent a structure from Basins B and C, respectively. In the central panel, the superimposition between the two latter structures is reported. When only one structure is reported, the ribbons are colored as in Figure 1C; in the superimposition, the structures from Basins B and C are in dark and light green, respectively. The Zn(II) and Mg(II) ions are shown as spheres, while the ADP molecules are shown as in sticks colored according to the atom type.

The conformational space revealed by the first two covariance eigenvectors was also inspected, and the resulting maps were used to search for significative sub-states of the conformational populations in the two systems. Figure 5A shows how the RNA-free form includes a region characterized by only one of the most visited conformations during the entire simulation (Basin A). The most representative structure in the absence of nucleic acid showed little difference from that identified by RMSD-based clustering (Figure 3A). On the other hand, a trajectory analysis of the RNA-engaged form showed two important differences from the previous case. First, two basins were noted, one narrow and highly populated (Basin B in Figure 5B), and the second less populated and wider (Basin C). The structures belonging to Basin C were observed in the first part of the simulation, and then evolved into the typical conformations of Basin B. The main difference between the representative structures of the two basins was in the conformation of the ZBD and 2A domains: the former bound more tightly to the rest of the protein, while the latter moved slightly away from the ZBD domain. The other domains did not undergo any significant changes. The RNA fragment did not undergo significant positional changes. The greater conformational variability (i.e., the greater amplitude of the single basin found) of the RNA-free form of NSP13 compared with the RNA-engaged form was in agreement with what has been seen in previous analyses.

#### 2.2.4. Comparison with Previous Simulations

The results presented in this manuscript are in excellent agreement with what has been obtained previously from a model of NSP13 from SARS-CoV-2 obtained by starting with the homologous protein from SARS-CoV and then inserting an ADP molecule and the nucleic acid fragment by modeling [26]. Our results show that on a five-fold time scale, using experimental structures and a state-of-the-art model for metal ions, very similar results were observed for the dynamics of NSP13’s domains. However, if one inserts the RNA fragment a posteriori, it is difficult to correctly model the movement of the 1B domain that is necessary to make room for the nucleic acid.

### 2.3. Interdomain Contact Frequencies

**Scheme 1:**
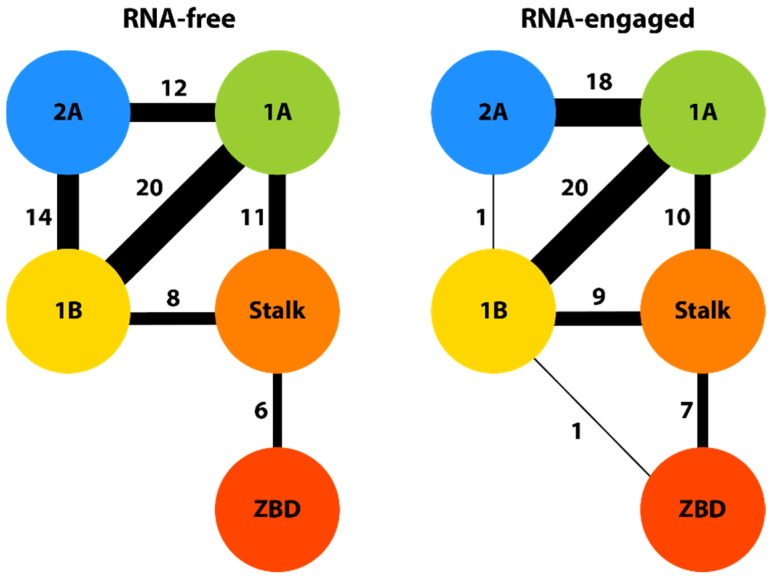
Number of contacts between the five NSP13 domains along the trajectory in the RNA-free (**left**) and RNA-engaged (**right**) forms. The domains are colored as in Figure 1C, while the thickness of the lines connecting the domains is proportional to the number of contacts. If no line connecting two domains is observed, then no contacts occurred during the simulation.

The contacts among the five NSP13 domains were analyzed by calculating inter-residue distance matrixes. In particular, the strategy adopted was to calculate the normalized inter-residue contact frequencies, defining two residues as being in contact when the distance between any two heavy atoms was less than 3.5 Å for at least the 50% of the simulation time. The emerging picture is reported in Scheme 1, where the number of interdomain contacts in the RNA-free and RNA-engaged NSP13 simulations is reported. The general emerging picture is that the absence of the RNA fragment closes the nucleic acid cavity between the 1B and 1A domains. Indeed, as noted earlier, Domain 1B is the one that changes orientation the most because of the presence of RNA. On the other hand, the presence of RNA reinforces the contacts between the 1A and 2A domains. The interactions between the pairs of domains (1B–2A and 1A–2A) in the two simulated forms of NSP13 are discussed in detail below, while the interactions between the other pairs of domains are given in the Supplementary Information (Figures S6–S10). Finally, an analysis of the interactions of the NSP13 residues in the RNA-engaged form with the RNA fragment and the ADP molecule was carried out.

#### 2.3.1. B–2A Interactions

**Figure 6:**
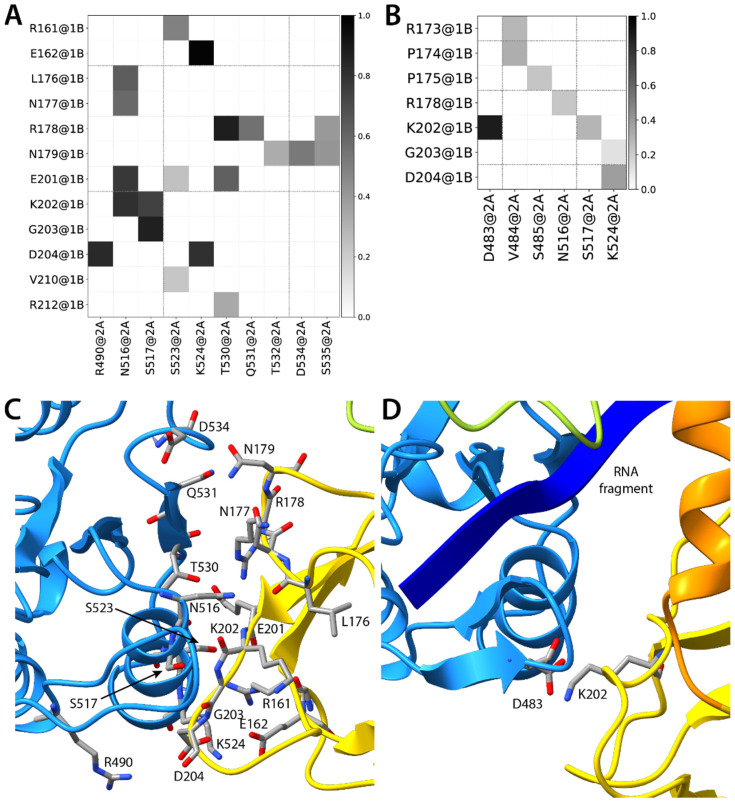
Plot of the interface contact matrix between Domains 1B and 2A in RNA-free (**A**) and RNA-engaged (**B**) NSP13. Contacts are colored from white to black if they were observed in 0% or 100% of the simulation time, respectively. In Panels (**C**) (RNA-free) and (**D**) (RNA-engaged), a detail of the 1B–2A interface taken from the representative structure of the most populated cluster is given. The ribbons are colored as in Figure 1C, while the residues involved in a contact lasting for at least 50% of the simulation time are reported as sticks colored according to the atom type.

The comparison of the interactions between the 1B and 1A domains (Figure 6) reflects the conformational displacement of the 1B domain upon RNA binding. Indeed, none of the numerous contacts detected in the RNA-free form was retained in the RNA-engaged form. In the RNA-free form, most of the interactions were H-bonds or salt bridges, while the only long-lasting contact in the RNA-engaged form (Lys202–Asp483) consisted of a salt bridge between the interacting side chains.

#### 2.3.2. A–2A Interactions

**Figure 7:**
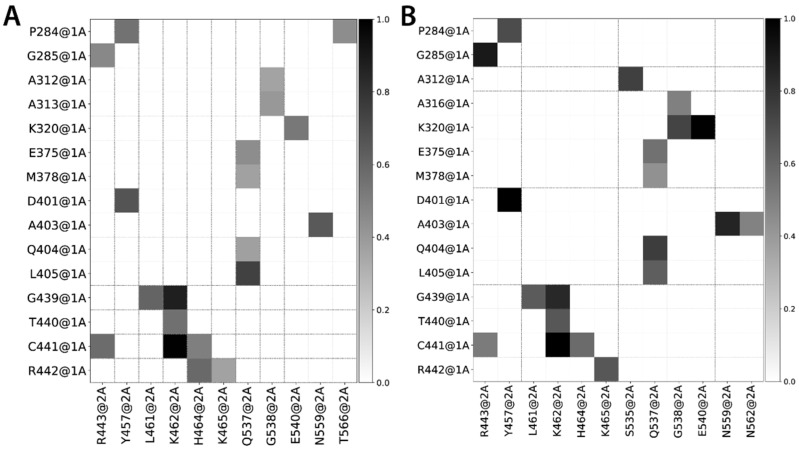
Plot of the interface contact matrix between Domains 1A and 2A in RNA-free (**A**) and RNA-engaged (**B**) NSP13. Contacts are colored from white to black if they were observed in 0% or 100% of the simulation time, respectively.

An analysis of the contacts between Domains 1A and 2A during the simulations (Figure 7) showed that the presence of RNA did not particularly perturb the interface between the two domains. The list of contacts was about the same in the RNA-free and RNA-engaged forms. However, in the RNA-engaged form, the contacts were in greater numbers, and interactions that were more labile in the RNA-free form became more stable and long-lasting.

#### 2.3.3. RNA–NSP13 Interactions

**Figure 8:**
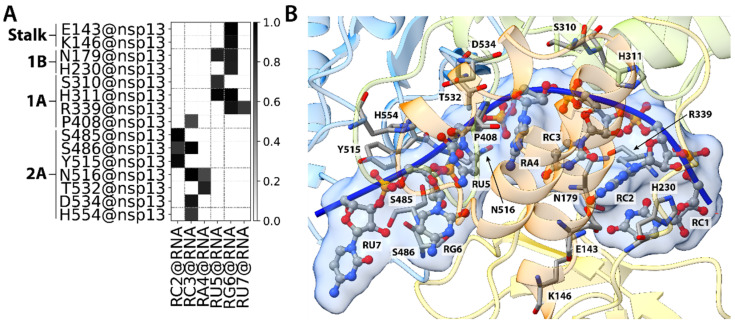
(**A**) Plot of the interface contact matrix between NSP13 and the RNA fragment present in the RNA-engaged form. Contacts are colored from white to black if they were observed in 0% or 100% of the simulation time, respectively. In Panel (**B**), a detail of the RNA–NSP13 interface taken from the representative structure of the most populated cluster is shown. The ribbons are colored as in Figure 1C, while the NSP13 residues involved in a contact lasting for at least 50% of the simulation time are reported as sticks, and the RNA nucleotides are shown as balls and sticks. All atoms are colored according to the atom type.

In the RNA-engaged form of NSP13, the RNA fragment appeared to establish several stable interactions with residues belonging to the Stalk, 1B, 1A, and 2A domains (Figure 8). The list of interactions did not change during the simulation, showing that the RNA fragment was not able to move into the channel at the microsecond timescale. This is consistent with the energy requirements (provided by ATP hydrolysis) required for RNA unwinding, which cannot be simulated by equilibrium molecular dynamics. The first nucleotide was fully exposed to the solvent and did not establish any interactions with NSP13. On the other hand, each of the remaining six nucleotides established one interaction with at least two NSP13 residues.

#### 2.3.4. ADP–NSP13 Interactions

Finally, the interactions of the ADP molecule present in the RNA-engaged form were also inspected. The ADP molecules formed extremely stable interactions (i.e., with a lifetime larger than 90% of the simulation time) with the nearby residues, which were Pro283, Pro284, Gly285, Thr286, Gly287, Lys288, His290, and Lys320 in the 1A domain, together with Arg443, Lys465, Glu540, and Lys569 in the 2A domain. The position and the coordination geometry of the Mg(II) ion was stable throughout the entire 10 µs of the simulation. The ADP molecule was bound in a pocket between Domains 1A and 2A, probably helping to stabilize the interaction between these two domains, in agreement with what was observed in Section 2.1.2 and Section 2.3.2

## 3. Materials and Methods

The model on which the simulations of the SARS-CoV-2 helicase in the RNA-free form were based was the crystallographic structure determined at a resolution of 1.94 Å with two subunits in the asymmetric unit (PDB ID 6ZSL). Generally, the electron density was of high quality, except for the lack of resolution of Residues 95–102, 186–193, 203–206, and 593–601 for Chain A and for Residues 1, 337–339, and 594–601 for Chain B. To correct these unresolved areas, homology modeling was performed using the Modeller 10.0 software [27] so Chain B included the residues not included in Chain A.

The starting structure for the helicase in the RNA-engaged form was the cryo-EM-resolved structure with a resolution of 3.10 Å of the SARS-CoV-2 replication–transcription complex bound to the NSP13 helicase-NSP13(2)-RTC-engaged class (PDB ID 7RDY). After this, only the helicase with the bound RNA fragment and the ADP molecule were retained.

The open-source web server H++ [28] was used to predict the protonation state of the proteins, based on the calculated pKa values of titratable groups. For the simulation, the Amber14sb force field [29] and TIP3P water model [30] were used for the protein and water molecules, respectively. For the helicase in the RNA-engaged form, the Mg(II) ion was treated by applying the optimized parameters described by Grotz et al. [31]. GAFF [32] ADP parameters were generated using the Antechamber software [33] included in the Amber 20 suite [34] and converted into GROMACS format by ParmEd. Zn(II) ions were treated by using the non-bonding parameters described by Macchiagodena et al. [35]. According to the same procedure, the Zn(II) binding cysteine and histidine residues were modified in order to account for the polarization effects of the cation.

The resulting structures were then placed in a truncated octahedral water box, where a solvent buffer zone of 10 Å was set by using the tools included in the GROMACS 2020.1 suite [36,37,38]. The systems were neutralized by adding Na^+^ ions by using the *genion* tool included in GROMACS 2020.1. Analogously, additional Na^+^ and Cl^−^ ions were placed in the water box to achieve physiological ionic strength (150 mM). The systems were energy-minimized with the “steepest descents” algorithms for 1500 steps and then equilibrated at 300 K and 1 atm by performing 1 ns of gradual annealing using GROMACS 2020.1. Next, to equilibrate the solvent around the protein, positional restraints were imposed on the protein, and each system was equilibrated for 50,000 steps with a time step of 2 fs at 300 K. The system’s pressure was coupled to a Berendsen [39] barostat, and the temperature was coupled to a V-rescale thermostat [40]. The LINCS algorithm [41] was used for imposing constraints on the hydrogen-containing bonds. Periodic boundary conditions (PBC) were applied and the Particle Mesh Ewald (PME) method [42] was used to calculate the electrostatic interactions. The cutoff values for the real part of the electrostatic interactions and for the van der Waals interactions were set to 10 Å. In the 10-μs-long production runs, the same protocol was adopted, with the exception of the use of the Parrinello–Raman barostat [43]. In a protein, interdomain movements not involving large conformational rearrangements usually occur on the sub-microsecond timescale [44,45]. For this reason, a simulation 10 μs long was deemed sufficient to provide the necessary information to study the dynamic behavior of NSP13’s domains in the RNA-free and RNA-engaged forms and the interdomain interactions. All calculations were performed at CINECA on the MARCONI100 accelerated cluster, based on nodes consisting of two IBM Power9 processors and four NVIDIA Volta GPUs per node. The dynamic performance on this architecture was typically 200 ns/day using 1 MARCONI100 node for each simulation.

The trajectories were analyzed by using GROMACS tools and in-house Python3 scripts, making use of the MDTraj 1.9.8 [46] and mdciao 0.0.5 [47] libraries. Covariance maps were visualized using the gmx_corr Python3 notebook [48]. The free energy maps were calculated as in [49]. The trajectories were visually inspected using NGLview 3.0.3 [50] and UCSF ChimeraX 1.3 [51,52].

## 4. Conclusions

In this work, extensive all-atom plain MD simulations were used to study the helicase (NSP13) of SARS-CoV-2 in its RNA-free form and RNA-engaged form. What emerged from the analyses was the main contribution of Domains 1A and 2A, the functional cores of the helicase, which make a major contribution to the stability and flexibility of the system of which they are part. Our simulations also highlighted the role of Domain 1B, which is responsible for forming the cavity in which the single RNA strand is lodged. The role of the ZBD domain as a domain supporting the functional activity of the helicase was also observed. The study showed a difference in the stability of ZBD domain between the RNA-free and RNA-engaged forms. As explained above, the ZBD is involved in the interaction with the other NSPs of the RTC. The differences highlighted during the simulations suggested that the presence of RNA can modify the dynamics of this domain, leading to the major stability of the interactions and promoting the activity of the complex itself. These results can be useful, not just to unveil the dynamics of NSP13 in the presence or absence of RNA, but also to define the main residues responsible for the stability of the system, representing putative regions to consider as targets for the development of new drugs. In particular, the NSP13 region defined by the residues found in contact with the RNA fragment (Figure 8) in the RNA-engaged form, as well as the residues mainly contributing to the interaction between the 1B and 2A domains in the RNA-free form (Glu162, Arg178, Gly203, and Asp204 from the 1B domain, and Lys524, Thr530, Ser517, Arg490, and Lys524 on the 2A domain) can be used in the search for putative binding pockets for the design of potential NSP13 inhibitors.

After these observations, the analyses moved on to a more structural level, investigating the interactions existing among the various domains in both systems under examination. The residues responsible for the domain–domain interactions were identified, outlining a profoundly different picture between NSP13 in the RNA-free and RNA-engaged forms. Looking ahead, it would be inspiring to run a molecular dynamics simulation of the unfolding of the RNA’s double helix to observe what the arrangement of the protein domains interacting with the RNA would be.

## Data Availability

The trajectories and the analyses are available upon request from the corresponding author.

## Supplementary Materials

The supporting information can be downloaded at. https://www.mdpi.com/article/10.3390/ijms232314721/s1.

## References

[1] Ciotti M., Angeletti S., Minieri M., Giovannetti M., Benvenuto D., Pascarella S., Sagnelli C., Bianchi M., Bernardini S., Ciccozzi M. (2019). COVID-19 Outbreak: An Overview. Chemotherapy.

[2] Hu B., Guo H., Zhou P., Shi Z.L. (2021). Characteristics of SARS-CoV-2 and COVID-19. Nat. Rev. Microbiol..

[3] Malone B., Urakova N., Snijder E.J., Campbell E.A. (2022). Structures and functions of coronavirus replication-transcription complexes and their relevance for SARS-CoV-2 drug design. Nat. Rev. Mol. Cell. Biol..

[4] Yoshimoto F.K. (2020). The Proteins of Severe Acute Respiratory Syndrome Coronavirus-2 (SARS-CoV-2 or n-COV19), the Cause of COVID-19. Protein J..

[5] Satarker S., Nampoothiri M. (2020). Structural Proteins in Severe Acute Respiratory Syndrome Coronavirus-2. Arch. Med. Res..

[6] Chan J.F., Kok K.H., Zhu Z., Chu H., To K.K., Yuan S., Yuen K.Y. (2020). Genomic characterization of the 2019 novel human-pathogenic coronavirus isolated from a patient with atypical pneumonia after visiting Wuhan. Emerg. Microbes Infect..

[7] Zhang J., Xiao T., Cai Y., Chen B. (2021). Structure of SARS-CoV-2 spike protein. Curr. Opin. Virol..

[8] Kung Y.A., Lee K.M., Chiang H.J., Huang S.Y., Wu C.J., Shih S.R. (2022). Molecular Virology of SARS-CoV-2 and Related Coronaviruses. Microbiol. Mol. Biol. Rev..

[9] Wolff G., Limpens R., Zevenhoven-Dobbe J.C., Laugks U., Zheng S., de Jong A.W.M., Koning R.I., Agard D.A., Grunewald K., Koster A.J. (2020). A molecular pore spans the double membrane of the coronavirus replication organelle. Science.

[10] V’Kovski P., Kratzel A., Steiner S., Stalder H., Thiel V. (2021). Coronavirus biology and replication: Implications for SARS-CoV-2. Nat. Rev. Microbiol..

[11] Hartenian E., Nandakumar D., Lari A., Ly M., Tucker J.M., Glaunsinger B.A. (2020). The molecular virology of coronaviruses. J. Biol. Chem..

[12] Hillen H.S., Kokic G., Farnung L., Dienemann C., Tegunov D., Cramer P. (2020). Structure of replicating SARS-CoV-2 polymerase. Nature.

[13] Yan L., Zhang Y., Ge J., Zheng L., Gao Y., Wang T., Jia Z., Wang H., Huang Y., Li M. (2020). Architecture of a SARS-CoV-2 mini replication and transcription complex. Nat. Commun..

[14] Wang Q., Wu J., Wang H., Gao Y., Liu Q., Mu A., Ji W., Yan L., Zhu Y., Zhu C. (2020). Structural Basis for RNA Replication by the SARS-CoV-2 Polymerase. Cell.

[15] Gao Y., Yan L., Huang Y., Liu F., Zhao Y., Cao L., Wang T., Sun Q., Ming Z., Zhang L. (2020). Structure of the RNA-dependent RNA polymerase from COVID-19 virus. Science.

[16] Romano M., Ruggiero A., Squeglia F., Maga G., Berisio R. (2020). A Structural View of SARS-CoV-2 RNA Replication Machinery: RNA Synthesis, Proofreading and Final Capping. Cells.

[17] Weber R., McCullagh M. (2021). Role of ATP in the RNA Translocation Mechanism of SARS-CoV-2 NSP13 Helicase. J. Phys. Chem. B.

[18] Chen J., Malone B., Llewellyn E., Grasso M., Shelton P.M.M., Olinares P.D.B., Maruthi K., Eng E.T., Vatandaslar H., Chait B.T. (2020). Structural Basis for Helicase-Polymerase Coupling in the SARS-CoV-2 Replication-Transcription Complex. Cell.

[19] Newman J.A., Douangamath A., Yadzani S., Yosaatmadja Y., Aimon A., Brandao-Neto J., Dunnett L., Gorrie-Stone T., Skyner R., Fearon D. (2021). Structure, mechanism and crystallographic fragment screening of the SARS-CoV-2 NSP13 helicase. Nat. Commun..

[20] Mickolajczyk K.J., Shelton P.M.M., Grasso M., Cao X., Warrington S.E., Aher A., Liu S., Kapoor T.M. (2021). Force-dependent stimulation of RNA unwinding by SARS-CoV-2 nsp13 helicase. Biophys. J..

[21] Jang K.J., Jeong S., Kang D.Y., Sp N., Yang Y.M., Kim D.E. (2020). A high ATP concentration enhances the cooperative translocation of the SARS coronavirus helicase nsP13 in the unwinding of duplex RNA. Sci. Rep..

[22] Chen J., Wang Q., Malone B., Llewellyn E., Pechersky Y., Maruthi K., Eng E.T., Perry J.K., Campbell E.A., Shaw D.E. (2022). Ensemble cryo-EM reveals conformational states of the nsp13 helicase in the SARS-CoV-2 helicase replication-transcription complex. Nat. Struct. Mol. Biol..

[23] White M.A., Lin W., Cheng X. (2020). Discovery of COVID-19 Inhibitors Targeting the SARS-CoV-2 Nsp13 Helicase. J. Phys. Chem. Lett..

[24] Savitzky A., Golay M.J.E. (1964). Smoothing and Differentiation of Data by Simplified Least Squares Procedures. Anal. Chem..

[25] Daura X., Gademann K., Jaun B., Seebach D., van Gunsteren W.F., Mark A.E. (1999). Peptide Folding: When Simulation Meets Experiment. Angew. Chem. Int. Ed..

[26] Perez-Lemus G.R., Menendez C.A., Alvarado W., Bylehn F., de Pablo J.J. (2022). Toward wide-spectrum antivirals against coronaviruses: Molecular characterization of SARS-CoV-2 NSP13 helicase inhibitors. Sci. Adv..

[27] Webb B., Sali A. (2016). Comparative Protein Structure Modeling Using MODELLER. Curr. Protoc. Bioinform..

[28] Anandakrishnan R., Aguilar B., Onufriev A.V. (2012). H++ 3.0: Automating pK prediction and the preparation of biomolecular structures for atomistic molecular modeling and simulations. Nucleic Acids Res..

[29] Maier J.A., Martinez C., Kasavajhala K., Wickstrom L., Hauser K.E., Simmerling C. (2015). ff14SB: Improving the Accuracy of Protein Side Chain and Backbone Parameters from ff99SB. J. Chem. Theory Comput..

[30] Jorgensen W.L., Chandrasekhar J., Madura J.D., Impey R.W., Klein M.L. (1983). Comparison of simple potential functions for simulating liquid water. J. Chem. Phys..

[31] Grotz K.K., Cruz-Leon S., Schwierz N. (2021). Optimized Magnesium Force Field Parameters for Biomolecular Simulations with Accurate Solvation, Ion-Binding, and Water-Exchange Properties. J. Chem. Theory Comput..

[32] Wang J., Wolf R.M., Caldwell J.W., Kollman P.A., Case D.A. (2004). Development and testing of a general amber force field. J. Comput. Chem..

[33] Wang J., Wang W., Kollman P.A., Case D.A. (2006). Automatic atom type and bond type perception in molecular mechanical calculations. J. Mol. Graph. Model..

[34] Case D.A., Cheatham III T.E., Darden T., Gohlke H., Luo R., Merz Jr K.M., Onufriev A., Simmerling C., Wang B., Woods R.J. (2005). The Amber biomolecular simulation programs. J. Comput. Chem..

[35] Macchiagodena M., Pagliai M., Andreini C., Rosato A., Procacci P. (2019). Upgrading and Validation of the AMBER Force Field for Histidine and Cysteine Zinc(II)-Binding Residues in Sites with Four Protein Ligands. J. Chem. Inf. Model..

[36] Berendsen H.J.C., van der Spoel D., van Drunen R. (1995). GROMACS: A message-passing parallel molecular dynamics implementation. Comput. Phys. Commun..

[37] Lindahl E., Hess B., van der Spoel D. (2001). GROMACS 3.0: A package for molecular simulation and trajectory analysis. Mol. Model. Annu..

[38] Van Der Spoel D., Lindahl E., Hess B., Groenhof G., Mark A.E., Berendsen H.J. (2005). GROMACS: Fast, flexible, and free. J. Comput. Chem..

[39] Berendsen H.J.C., Postma J.P.M., van Gunsteren W.F., DiNola A., Haak J.R. (1984). Molecular dynamics with coupling to an external bath. J. Chem. Phys..

[40] Bussi G., Donadio D., Parrinello M. (2007). Canonical sampling through velocity rescaling. J. Chem. Phys..

[41] Hess B., Bekker H., Berendsen H.J.C., Fraaije J.G.E.M. (1997). LINCS: A linear constraint solver for molecular simulations. J. Comput. Chem..

[42] Essmann U., Perera L., Berkowitz M.L., Darden T., Lee H., Pedersen L.G. (1995). A smooth particle mesh Ewald method. J. Chem. Phys..

[43] Parrinello M., Rahman A. (1981). Polymorphic transitions in single crystals: A new molecular dynamics method. J. Appl. Phys..

[44] Guo J., Zhou H.X. (2016). Protein Allostery and Conformational Dynamics. Chem. Rev..

[45] Wei G., Xi W., Nussinov R., Ma B. (2016). Protein Ensembles: How Does Nature Harness Thermodynamic Fluctuations for Life? The Diverse Functional Roles of Conformational Ensembles in the Cell. Chem. Rev..

[46] McGibbon R.T., Beauchamp K.A., Harrigan M.P., Klein C., Swails J.M., Hernández C.X., Schwantes C.R., Wang L.-P., Lane T.J., Pande V.S. (2015). MDTraj: A Modern Open Library for the Analysis of Molecular Dynamics Trajectories. Biophys. J..

[47] Pérez-Hernández G., Tiwari S. (2021). gph82/mdciao: First Stable Release Candidate.

[48] Berçin Barlas A., Savaş B., Karaca E. (2022). CSB-KaracaLab/gmx_corr.

[49] Toba S., Colombo G., Merz K.M. (1999). Solvent Dynamics and Mechanism of Proton Transfer in Human Carbonic Anhydrase II. J. Am. Chem. Soc..

[50] Nguyen H., Case D.A., Rose A.S. (2018). NGLview–interactive molecular graphics for Jupyter notebooks. Bioinformatics.

[51] Goddard T.D., Huang C.C., Meng E.C., Pettersen E.F., Couch G.S., Morris J.H., Ferrin T.E. (2018). UCSF ChimeraX: Meeting modern challenges in visualization and analysis. Protein Sci..

[52] Pettersen E.F., Goddard T.D., Huang C.C., Meng E.C., Couch G.S., Croll T.I., Morris J.H., Ferrin T.E. (2021). UCSF ChimeraX: Structure visualization for researchers, educators, and developers. Protein Sci..

