# Supplementary material for: Computational Study of Helicase from SARS-CoV-2 in RNA-Free and Engaged Form
Source: Int J Mol Sci. 2022 Nov 25;23(23):14721. doi: 10.3390/ijms232314721 (PMC9738952; doi:10.3390/ijms232314721)
Supplement: Supplementary file 1 [file ijms-23-14721-s001.zip › ijms-2029536-supplementary.pdf]

Computational study of helicase from SARS-CoV-2 in RNA-free and engaged form

Francesca Di Matteo,[1] Giorgia Frumenzio,[2] Balasubramanian Chandramouli,[2] Alessandro Grottesi,[3] Andrew Emerson,[2] and Francesco Musiani[1,*]

1 Laboratory of Bioinorganic Chemistry, Department of Pharmacy and Biotechnology, University of Bologna, Viale G. Fanin 40, Bologna, 40127, Italy.

2 Super Computing Applications and Innovation, Department HPC, CINECA, via Magnanelli 6/3, Casalecchio di Reno, 40033, BO, Italy.

3 Department HPC, CINECA, via dei Tizii 6, Roma, 00185, Italy.

* Corresponding author: francesco.musiani@unibo.it

SUPPLEMENTARY INFORMATION

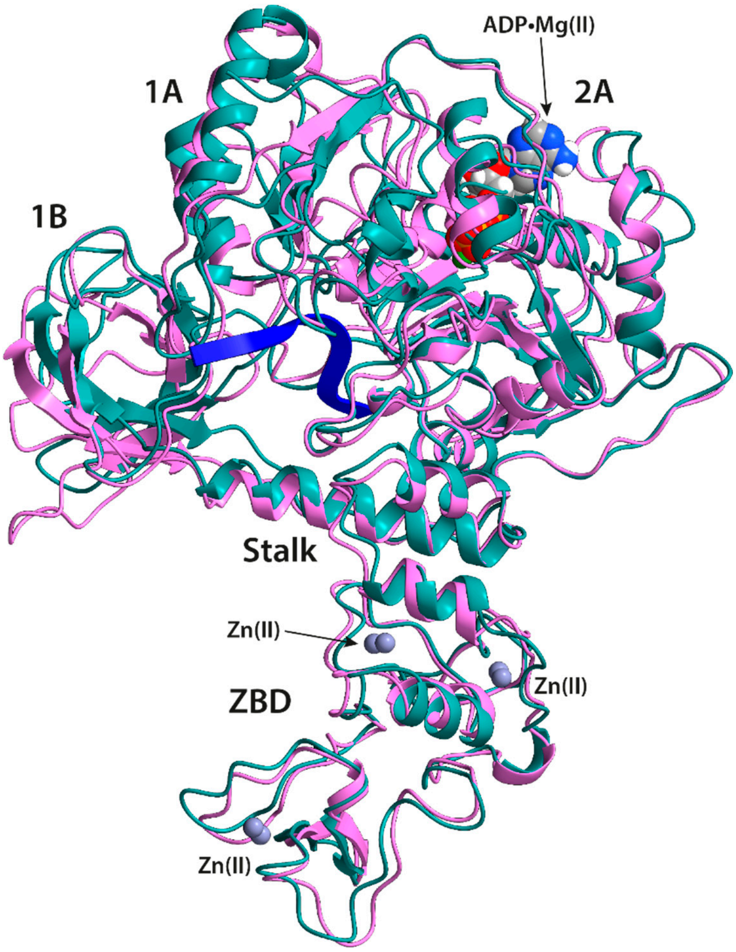

Figure S1. Superimposition of NSP13 initial structures in the RNA-free (dark cyan ribbons) and RNA-engaged (orchid ribbons) form. The RNA fragment is reported as a blue ribbon. The Zn(II) and Mg(II) ions as well as the ADP molecule are reported as spheres colored according to the atom type.

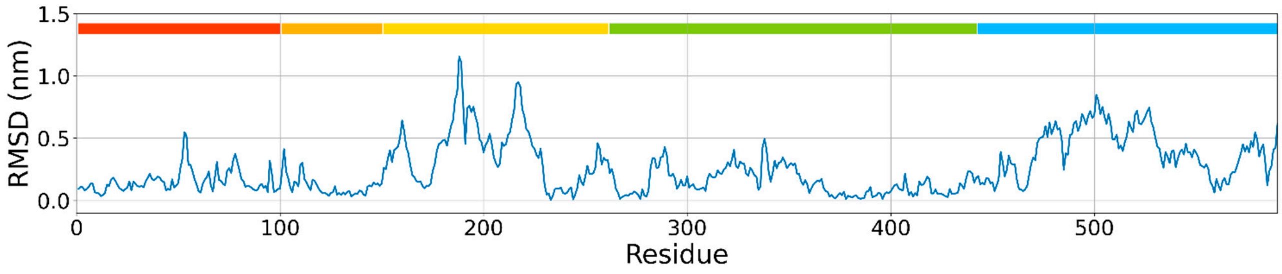

Figure S2. Calculated per residue Cα root mean square deviations (RMSD) of NSP13 between the RNA-free and RNA-engaged initial structures. The horizontal bars report the position of each domain in the sequence and are colored according to the domain coloration in Figure 1C.

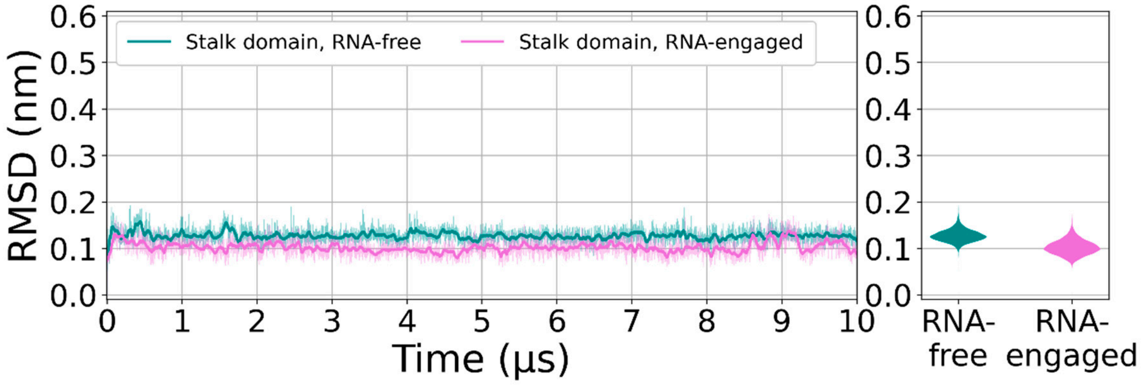

Figure S3. Calculated root mean square deviations (RMSD) of Stalk domain from the initial structures plotted as a function of the simulation time. RMSD values for Stalk domain in the RNA-free and in the RNA-engaged form are in dark cyan and orchid, respectively. The bold lines are obtained by applying a Savitzky-Golay filter in order to cut-off the noise. In the right panel, the violin representation of the RMSD distribution is provided.

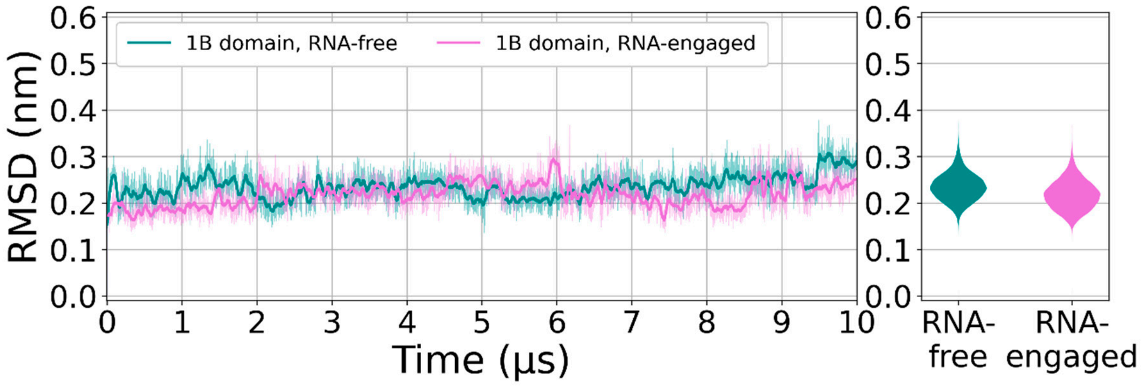

Figure S4. Calculated root mean square deviations (RMSD) of 1B domain from the initial structures plotted as a function of the simulation time. RMSD values for 1B domain in the RNA-free and in the RNA-engaged form are in dark cyan and orchid, respectively. The bold lines are obtained by applying a Savitzky-Golay filter in order to cut-off the noise. In the right panel, the violin representation of the RMSD distribution is provided.

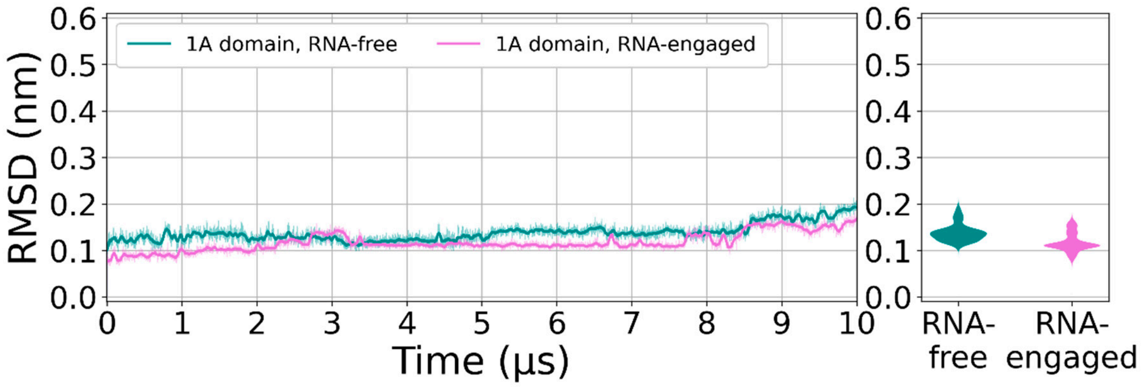

Figure S5. Calculated root mean square deviations (RMSD) of 1A domain from the initial structures plotted as a function of the simulation time. RMSD values for 1A domain in the RNA-free and in the RNA-engaged form are in dark cyan and orchid, respectively. The bold lines are obtained by applying a Savitzky-Golay filter in order to cut-off the noise. In the right panel, the violin representation of the RMSD distribution is provided.

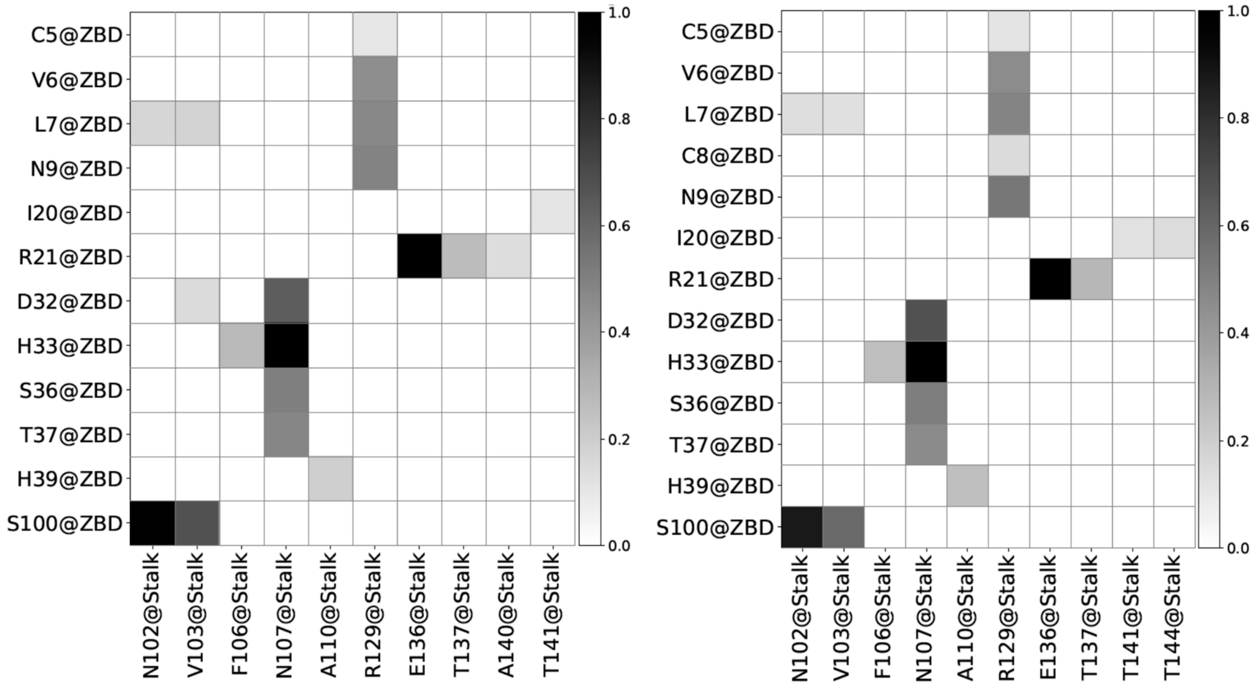

Figure S6. Plot of the interface contact matrix between domains ZBD and Stalk in the RNA-free (left panel) and RNA-engaged (right panel) nsp13. Contacts are colored from white to black if they are observed in the 0% or 100% of the simulation time, respectively.

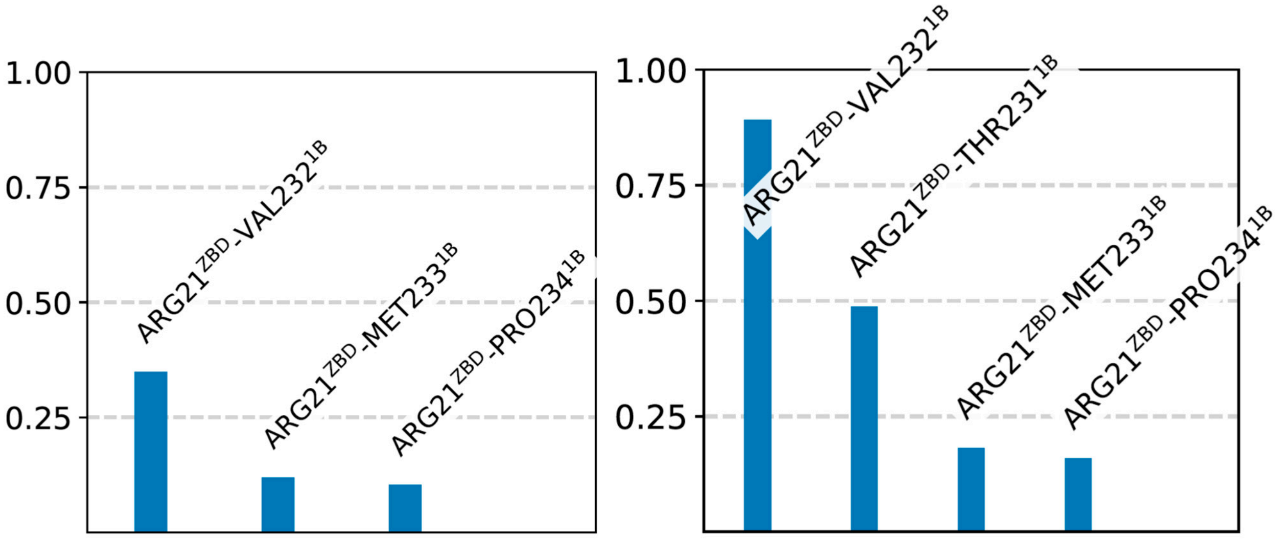

Figure S7. Contact frequencies between domains ZBD and 1B in the RNA-free (left panel) and RNA-engaged (right panel) nsp13. Bar heights are proportional to the relative contact time observed during the simulations.

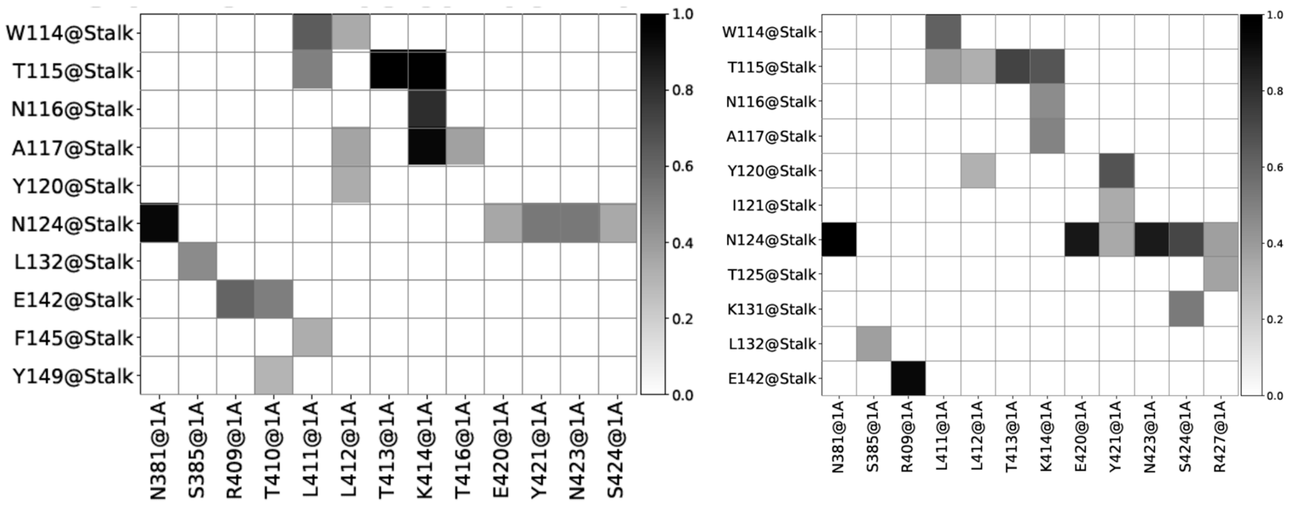

Figure S8. Plot of the interface contact matrix between domains Stalk and 1A in the RNA-free (left panel) and RNA-engaged (right panel) nsp13. Contacts are colored from white to black if they are observed in the 0% or 100% of the simulation time, respectively.

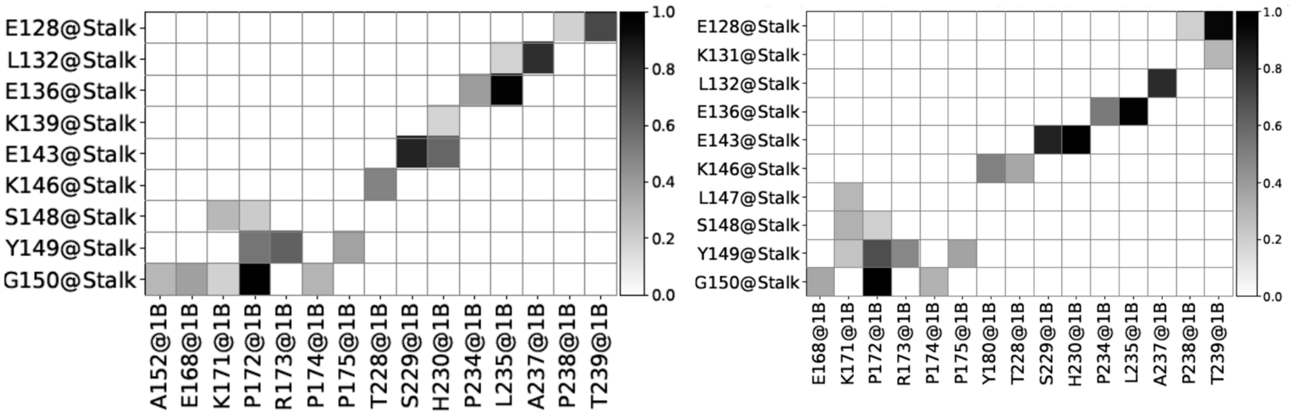

Figure S9. Plot of the interface contact matrix between domains Stalk and 1B in the RNA-free (left panel) and RNA-engaged (right panel) nsp13. Contacts are colored from white to black if they are observed in the 0% or 100% of the simulation time, respectively.

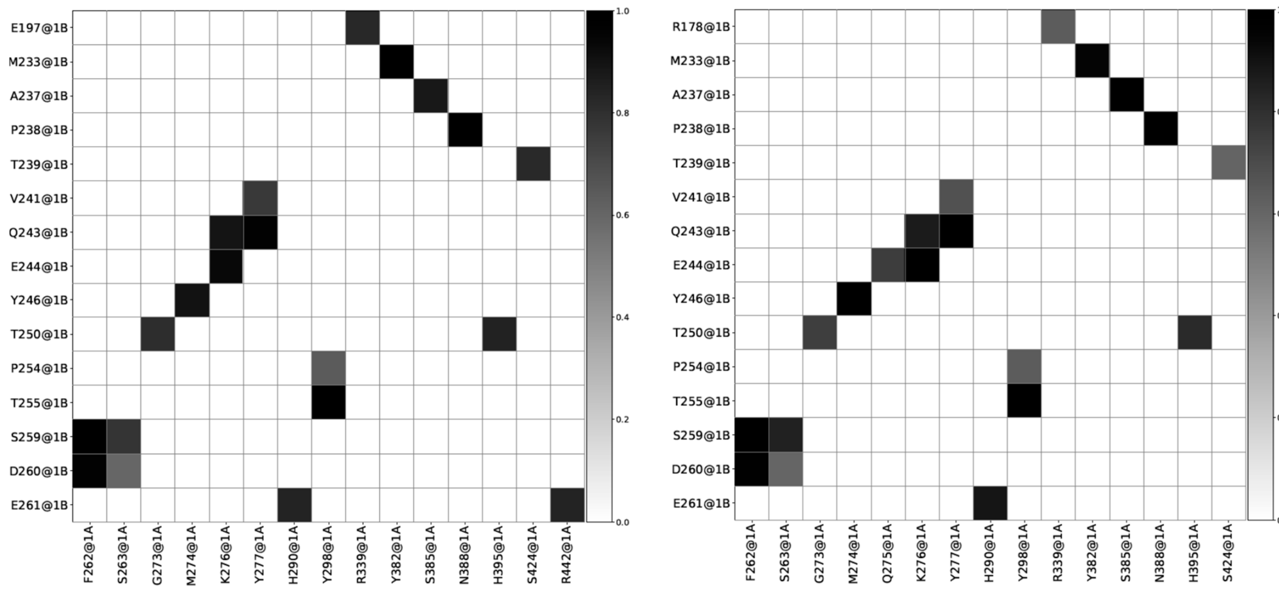

Figure S10. Plot of the interface contact matrix between domains 1A and 1B in the RNA-free (left panel) and RNA-engaged (right panel) nsp13. Contacts are colored from white to black if they are observed in the 0% or 100% of the simulation time, respectively.
